# Catalogue of the Surgical and Pathological Museum of Valentine Mott, M.D.

**Published:** 1859-03

**Authors:** 


ART. V.—*Catalogue of the Surgical and Pathological Museum of* VALENTINE MOTT, M.D., LL.D., Professor of Surgery in the University of the City of New York, etc., and of his son, ALEXANDER B. MOTT, M.D., etc. 8vo., pp. 78. New York, 1858.

As will be perceived from its title, this pamphlet comprises an enumeration of the many surgical preparations and specimens of morbid and normal anatomy contained in the collection of Dr. Mott, a name as familiar as household words to every medical man on both sides of the Atlantic, as having contributed more to the honor and advancement of the science of surgery in this country than any of its worthy sons. His operations upon the arteries alone would have been sufficient to have made him an enviable reputation, had he done nothing more; but when we add to this his valuable contributions to surgical literature, his untiring professional zeal even during his declining years, and his numerous and varied operations, we can but regard him as a most extraordinary man. As a general rule, the specimens are simply enumerated in the order in which they occur in the various compartments of the museum; to some, however, histories are attached, which render them valuable not only to the students of the University, for whom the work is especially designed, but to the practitioner who cannot be familiar with the cases, as they were, for the most part, published in medical periodicals many years ago. A point of interest to the surgical pathologist is that in all the cases of spontaneous gangrene here reported, it will be found from the histories of the cases that the main artery of the limb was obliterated either by ossific or fibrinous deposits. In speaking of a specimen of ulcerated carcinoma of the heart, Dr. Mott observes that he has never seen a case in which, after its removal, the disease did not recur; an opinion in favor of which the great mass of the profession can bear testimony. Young surgeons of the present day would do well to bear this point in mind before operating.

The museum abounds in specimens of cancer in its various types, and we are struck with the frequency with which the bones are affected. In this connection we may allude to one of Dr. Mott's most difficult operations, that of removal of the clavicle for osteo-sarcoma. In this case not less than forty ligatures were applied to bleeding vessels, among which was included the external jugular vein. The patient lost but comparatively little blood, and made a good recovery at the expiration of two months. The ligation of the external jugular is interesting when taken in connection with several operations here described, in which large venous trunks required tying, as the femoral and axillary veins, without any bad consequences. The greatest of all Dr. Mott's operations is that of ligation of the innominate artery, which he was the first to perform. Inasmuch as the case has been out of print for some time, and on account of its great interest, we do not deem it necessary to apologize for inserting it for the benefit of any of our readers who may not be familiar with its details:—

"*Ligature of the Innominata.*—The pioneer operation on the Arteria Innominata. It was performed on Michael Bateman, a seaman, twenty-seven years of age, in the New York Hospital, May 11, 1818. About a week previous to entering the hospital, while at work on shipboard, he slipped and fell on the shoulder of that side, and in two or three days it was so much swollen as to incapacitate him from duty.

"March 1st, he entered the hospital as a medical patient, for a catarrhal affection, which at that time attracted most attention. The shoulder, however, was blistered repeatedly, under which treatment the swelling somewhat subsided, leaving the tumor more distinct and circumscribed, and revealing an obscure pulsation. This was at first supposed to be communicated by the subclavian artery, as the tumor was hard and unyielding.

"May 3d, he felt 'something give way' in it, and the enlargement suddenly increased, fully one-third, with much pain in the shoulder and inability to raise the arm more than a few inches from the side. As the pulsation was now distinctly perceptible, though the firmness remained, he was transferred to the surgical side of the house.

"After due consultation with Drs. W. Post, Kissam, and Stevens, and with their concurrence, I resolved to attempt the ligature of the subclavian artery within the scalenus muscle. If the size of the aneurism, however, should be found to prevent this, as a dernier measure the brachio cephalic trunk itself was to be tied. Dr. Post made a full and candid explanation to him of the nature and probable termination of his disease and of the uncertain character of the operation, after which he requested to have it performed as furnishing him some chance of life.

"May 11th the tumor had become so irregular in form that it is difficult to give an idea of its size. It had elevated the clavicle, by its pressure, about an inch above the level of the opposite bone, and it further rose above the bone in the form of two lobes, fully another inch. A thread crossing it, obliquely, upward and inward, toward the back of the neck, from margin to margin, measured five and a quarter inches. A second thread, crossing the first at right angles, an inch above the clavicle, measured four inches—two and a half inches being on the sternal side and one and a half on the acromial. His pulse was sixty-nine and regular, with no difference at the two wrists.

"After placing him in the recumbent posture on the table, an incision about three inches long was made just above the clavicle and parallel to that bone, terminating over the trachea, at which point it was met by a second incision of about the same length, along the inner edge of the sterno-cleidomastoid; the two making a V, the angle of which was reflected from the platysma myoides, and this muscle divided. All the sternal portion of the mastoid muscle was then divided as well as the greater part of the caviclar portion, in order to turn it upward and outward. The latter portion proved to be morbidly adherent to the internal jugular vein, requiring the utmost delicacy in its detachment, and limiting the room for the subsequent steps of the operation. The sterno-hyoid and sterno-thyroid muscles were then divided and reflected in the opposite direction, over the trachea. The sheath of the jugular vessels was now opened near the sternum, and the nerve and vein drawn to the outside, while the artery was pressed toward the trachea. About half an inch of the subclavian artery was then brought into view, which appeared considerably enlarged and of an unhealthy color, so that my friends concurred with me in the impropriety of passing a ligature around it. In fact, the close proximity of the tumor was of itself sufficient reason for no ligature here, as healthy adhesive inflammation was not to be anticipated in the vicinity of so much disease.

"Proceeding downward toward the innominata, when about half an inch above it, while cautiously separating the artery with the smooth ivory handle of the scalpel, a small branch, about the size of a crow's quill, was lacerated, which filled the wound, perhaps six or eight times, but was stopped by pressure. The bifurcation exposed, the dissection was further prosecuted behind the sternum, and finally the brachio cephalic trunk isolated from the pleura. This was done with the handle of the knife, to prevent wounding that membrane. By means of a small blunt aneurism-needle the ligature was then passed from below upward, and a knot formed and gradually tightened by means of the two forefingers introduced into the wound.

"I acknowledge it was with feelings of intense anxiety, and with my eyes fixed on his countenance, that I ventured, for the first time in the annals of surgery, to intercept one-fourth of the whole blood so close to the heart. I drew the ligature gradually, determined to loosen it instantly if any alarming symptoms appeared. But there was no change of feature or agitation of body, and with the highest gratification I continued to draw the knot, until the circulation in this great trunk was completely arrested. This was shown by cessation of pulsation in the arteries of the right wrist and temple, as well as diminution by a third or more in the size of the tumor. Ten minutes after the operation the pulse was again beating regularly, sixty-nine in a minute. Only three small arteries were tied.

"There followed but little diminution of temperature in the right arm. The pulse in the evening being strong and full, and about seventy-five, he was bled xvi. oz. On the second day, in the afternoon, as it had again become tense, with other symptoms of febrile reaction, he was again bled viii. oz. and ordered light diet and salines. At the end of ten days it was about one hundred and ten, from which time it again diminished in frequency to a healthy standard. On the fourteenth day the large ligature which had been confined over the sternum with a strip of adhesive plaster, came away. From this time he continued to improve, and had become able to walk about the wards and the garden of the hospital, when, on the twenty-third day he was suddenly seized with an alarming hemorrhage from the wound, reducing him to syncope before it abated. All attempts to prevent recurrence of the bleeding, by stuffing the wound, proved ineffective, and he perished on the twenty-sixth day of exhaustion.

"On post-mortem examination the ulcer was found at the bottom twice the size of the wound in the neck. It communicated freely above with the subclavian and carotid arteries, and below by a small opening with the innominata. The latter artery was partially plugged by a coagulum, which extended lowest on the side opposite the anomalous branch. The common carotid was so thickly lined by coagulum that a probe could scarcely be introduced; not only to its division but beyond. The subclavian, however, was pervious throughout, as well as the arteries of the arm. The external mammary arteries, where they leave the axillary, were enlarged; the internal mammary was natural. The clavicle was found carious and entirely disunited about the middle. A number of lymphatic glands were in a state of scrofulous suppuration.

"The separation of the ligature in this case on the fourteenth day, spontaneously, and without hemorrhage for a number of days, conclusively proves to my mind that all the purposes of ligature were completely answered; that adhesion was fully effected, and that the ulceration, which went on so insidiously at the bottom of the wound, while the upper part appeared healing, was the sole cause of the death of my patient.

"For full description of the operation and post-mortem examination, see *Hospital Register*, vols. i. and ii.; also, *Velpeau's Oper. Surg.*, Am. ed."

Quite a number of the specimens contained in this collection belong to and are the result, in many instances, of the operations of Dr. A. B. Mott. Had the arrangement of the specimens been made in a more systematic manner, the catalogue would have been of more value and usefulness, not only to the student, but to the general reader.

